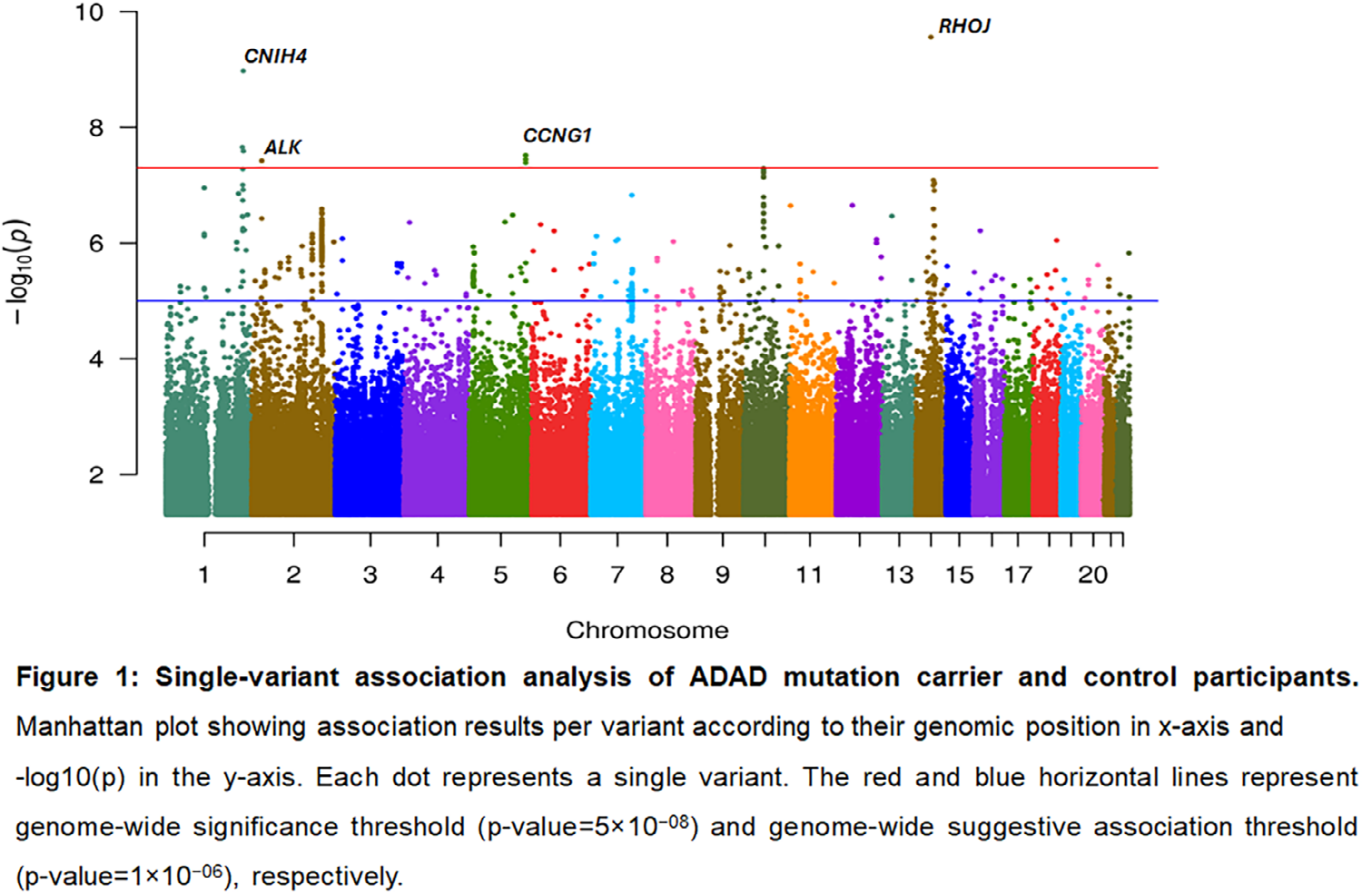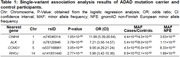# Identification of genetic modifiers influencing disease risk in autosomal dominant Alzheimer disease mutation carriers

**DOI:** 10.1002/alz70855_105550

**Published:** 2025-12-24

**Authors:** Maulikkumar P Patel, Arda Cetin, Matthew Johnson, Natalie S Ryan, John P. Budde, Menghan Liu, Jorge J. Llibre‐Guerra, John C. Morris, Randall J. Bateman, David M. Holtzman, Laura Ibanez, Alison M. Goate, Alan E. Renton, Carlos Cruchaga, Cyril P Pottier, Dominantly Inherited Alzheimer Network (DIAN)

**Affiliations:** ^1^ Department of Psychiatry, Washington University School of Medicine, St. Louis, MO, USA; ^2^ NeuroGenomics and Informatics Center, Washington University School of Medicine, St. Louis, MO, USA; ^3^ Dementia Research Centre, UCL Queen Square Institute of Neurology, London, United Kingdom; ^4^ UK Dementia Research Institute at UCL, London, United Kingdom; ^5^ Department of Neurology, Washington University School of Medicine, St. Louis, MO, USA; ^6^ Hope Center for Neurological Disorders, Washington University School of Medicine, St. Louis, MO, USA; ^7^ The Charles F. and Joanne Knight Alzheimer Disease Research Center, St. Louis, MO, USA; ^8^ Hope Center for Neurological Disorders, Washington University in St. Louis, St. Louis, MO, USA; ^9^ Knight Alzheimer Disease Research Center, St. Louis, MO, USA; ^10^ Nash Family Department of Neuroscience, Icahn School of Medicine at Mount Sinai, New York, NY, USA; ^11^ Ronald M Loeb Center for Alzheimer's Disease, Department of Genetics & Genomic Sciences, Icahn School of Medicine at Mount Sinai, New York, NY, USA; ^12^ Department of Genetics and Genomic Sciences, Icahn School of Medicine at Mount Sinai, New York, NY, USA; ^13^ Washington University School of Medicine, St. Louis, MO, USA; ^14^ The Charles F. and Joanne Knight Alzheimer Disease Research Center, St Louis, MO, USA; ^15^ Department of Psychiatry, Washington University in St. Louis School of Medicine, St. Louis, MO, USA

## Abstract

**Background:**

Alzheimer disease participants with autosomal dominant mutations in PSEN1, PSEN2, and APP (ADAD) exhibit variability in clinical presentation (age at onset and clinical symptoms), even within the same family. We hypothesized that even though ADAD participants carry highly penetrant mutations, additional and more common genetics variants may influence the risk of developing the disease. Therefore, we aimed to identify genetic modifiers of disease risk in ADAD mutation carriers using a case/control approach.

**Method:**

We conducted a genome‐wide association study assessing the effect of common genetic variants (minor allele frequency <0.005) on AD risk using a logistic regression model. Our cohort included whole genome sequencing data from 101 unrelated symptomatic participants with ADAD mutations and 5,050 asymptomatic unrelated control participants without ADAD mutations of non‐Hispanic European ancestry. Participants were recruited through the Charles F. and Joanne Knight Alzheimer Disease Research Center, Alzheimer's Disease Sequencing Project, and Dominantly Inherited Alzheimer Network. To assess the effect of the newly identified loci, we performed age at onset analysis using a linear regression model, and Kaplan‐Meier survival analysis. Segregation analyses were conducted within families. Finally, we conducted linear regression analyses on cerebrospinal fluid biomarkers (Aβ42/40, tTau, pTau181) obtained with Lumipulse assay for 91 participants. All analyses were adjusted for sex, 10 first principal components and cohort when appropriate.

**Results:**

We identified four genome‐wide significant loci associated with AD risk (Table 1, Figure 1). Remarkably, the association signal at the CNIH4 locus was driven by a missense variant (p.Gly54Ser). The rare allele at the CCNG1 locus reduced the age at AD onset (*p*‐value=6.81×10^‐03^, beta=‐10.15). This finding was further confirmed using Kaplan‐Meier analysis (*p*‐value=9.3×10^‐04^) with a median age at onset was 48 for non‐carriers and 40 for carriers. The rare allele was consistently present in participants with an earlier age at onset in two independent families. Finally, at the RHOJ locus, the rare allele was associated with increased tTau (*p*‐value=4.74×10^‐05^) and pTau181 (*p*‐value=4.07×10^‐07^), and decreased Aβ42/40 ratio (*p*‐value=4.32×10^‐05^).

**Conclusion:**

We identified 4 genetic modifiers that influence disease risk and affect multiple ADAD mutations, offering insights for future clinical trials and genetic counseling.